# The Beneficial or Harmful Effects of Computer Game Stress on Cognitive Functions of Players

**DOI:** 10.29252/nirp.bcn.9.3.177

**Published:** 2018

**Authors:** Hamed Aliyari, Hedayat Sahraei, Mohammad Reza Daliri, Behrouz Minaei-Bidgoli, Masoomeh Kazemi, Hassan Agaei, Mohammad Sahraei, Seyed Mohammad Ali Seyed Hosseini, Mohammad Mehdi Hadipour, Mohammad Mohammadi, Zahra Dehghanimohammadabadi

**Affiliations:** 1. Department of Electrical Engineering, Faculty of Electrical, Biomedical and Mechatronics Engineering, Qazvin Branch, Islamic Azad University, Qazvin, Iran.; 2. Neuroscience Research Center, Baqiyatallah University of Medical Sciences, Tehran, Iran.; 3. Department of Biomedical Engineering, Faculty of Electrical Engineering, Iran University of Science and Technology, Tehran, Iran.; 4. Department of Computer Engineering, School of Computer Engineering, Iran University of Science and Technology, Tehran, Iran.; 5. Department of Dentistry, Faculty of Dentistry, Shahid Beheshti University of Medical Sciences, Tehran, Iran.; 6. Department of Social Sciences, North Tehran Branch, Islamic Azad University, Tehran, Iran.; 7. Human Motion Control and Computational Neuroscience Laboratory, School of Electrical and Computer Engineering, College of Engineering, University of Tehran, Tehran, Iran.; 8. Department of Statistics, Faculty of Mathematical Sciences, Alzahra University, Tehran, Iran.

**Keywords:** Stress, Puzzle game, Runner game, Excitement game, Fear game, NeuroGame

## Abstract

**Introduction:**

Video games are common cultural issues with great influence in all societies. One of the important cognitive effects of video games is on creating stress on video players. The present research objective was to study different types of stress in players based on video game styles.

**Methods:**

A total of 80 players, aged 18 to 30 years, played four types of video games; Runner game, Excitement game, Fear game, and Puzzle game. In the beginning, the players filled in the form of personal information as well as some general and specialized information on the games. Before starting each game, the saliva samples of the players were collected to measure their level of cortisol and α-amylase. At the end of each game, the same samples were collected again. The concentrations of cortisol and α-amylase were measured using a specialized kit and an ELISA device. In addition, the variations of brain waves were recorded by an Emotiv system. Finally, the data were analyzed in SPSS and Matlab system (after and before playing video game).

**Results:**

The research findings revealed that the salivary α-amylase concentration increased significantly after playing the Fear game, Runner game, and Excitement game and decreased significantly after playing the Puzzle game. Moreover, the concentration of salivary cortisol increased significantly after playing the Runner game, Excitement game, and Fear game and decreased significantly after playing the Puzzle game. The brain wave analysis also revealed that the level of stress experienced by playing Fear game was higher than the Excitement game.

**Conclusion:**

According to the research findings, video games can affect the stress system as well as the cognitive system of humans depending on the game style. In addition, the type and level of stress triggered in the players depend on the game style.

## Highlights

- Video games have different effects on the stress of players.
- The type of stress depends on the game style.
- The resulting stress may reinforce the cognitive elements or destroy them.

## Plain Language Summary

The attractiveness of the computer games and also the day by day increase on the number of their corresponding audiences on one hand, and their different cognitive (positive and negative) effects on the players’ nervous system on the other hand have motivated conducting research and investigation in this area. The purpose of this research is to study the level and type of different stress effects on the players based on the game style diversity. In this research, four styles of computer games were studied: Puzzle games, Runner games, Excitement games, and Fear games. Based on the results, four different types of stress have been found from the NuroGame perspective. In other words, we studied the possibility of categorizing the games on the basis of their stress effect on players and the classification was made based on that. The results have depicted different cognitive responses and alterations on the players (in positive and negative ways) that makes the classification possible.

## 1. Introduction

Video games are an interactive medium and a novel technology spreading all over the globe. Neurologists and researchers are much interested in video games due to their effects on the nervous system. Because of their nature, these games can activate different parts of the brain. They activate the cortical regions of the brain such as the frontal cortex, the sensory-motor cortex, the visual cortex, and the subcortical areas (such as the amygdala and hippocampus), which play a substantial role in emotions and cognition, and thus considerably contribute to the personal behavior and cognitive functions (Aliyari et al., 2015; Lupien et al., 2005; Smeets, Otgaar, Candel, & Wolf, 2008). Stress is an important cognitive reaction significantly affected by video games.

According to new definitions, stress is a mental state affecting both mind and body. Some parts of the brain such as the prefrontal lobe are mainly associated with cognition and stress experience. Stress is of the acute and chronic types. Acute stress triggers the autonomic nervous system and increases the excretion of the cortisol, adrenaline, and other hormones (Lupien et al., 2005; Smeets et al., 2008). The unnatural rise in the level of cortisol results in an increase in the heartbeat, respiration rate, and blood pressure. Accordingly, the blood redirects from the end organs to the large muscles so the body gets ready to fight or escape. This condition is known as the fight-or-flight response. Chronic stress, on the other hand, refers to the long-term effect of one or several stress factors on human’s life. These chronic stress stimulations may result in the outbreak of neurological diseases such as anxiety and depression (Beylin & Shors, 2003; Lupien & Lepage, 2001; McEwen & Morrison, 2013).

Concerning neuroanatomical positions, many parts of the brain are associated with the stress state. Some of these areas include hypothalamus, hippocampus, amygdala, prefrontal cortex as well as forebrain and midbrain, which are associated with chronic stress and the down-scaling of the dendrites (Krawczyk, 2002; McEwen & Morrison, 2013; Rosenbloom, Schmahmann, & Price, 2012). The pathologic activation of the stress system and the adrenal-sympathetic system leads to an unnatural increase of the hormones and impairment of the nervous system. In this regard, video games are among the important causes of changes of the cognitive functions (Aliyari et al., 2015; Bartholow, Bushman, & Sestir, 2006; Lupien et al., 2005). Since the stress system is closely associated with cognitive functions (i.e. memory, learning, and reward) in the nervous system, the secretion of cortisol has different dose-dependent effects. An effective dose of cortisol enables humans to cope with stress and plays an important role in consciousness, motivation, attention, concentration, memory, decision making, and learning. However, an improper dose results in the inability to cope with stress, affects the normal performance of these systems, and impairs the order and coordination of the brain functions (i.e. concentration, attention, decision making, memory, and learning). Hence, the person’s cognition may be impaired under chronic stress (Anderson, Kludt, & Bavelier, 2011; Beylin & Shors, 2003; Lupien et al., 2005; Maheu, Joober, & Lupien, 2005).

Research results have indicated that improper performance of the stress system may result in reactions generally known as fear. One of the most important responses to this malfunction is the increase in the secretion of the salivary enzyme (α-amylase). This reaction happens very quickly and shows its effect within a few seconds. Hence, an increase in the concentration of the salivary α-amylase is a noninvasive simple bio-indicator that could be used to measure the activity of the sympathetic nervous system. In other words, this enzyme is known as the fear marker (Davis & Granger, 2009; Feinstein, Adolphs, Damasio, & Tranel, 2011; Nater & Rohleder, 2009), in other words, a valuable means of studying the stress-induced fear. Research results have indicated that the level of this enzyme varies in response to acute and chronic stress. The increase in the level of this enzyme occurs due to an increase in the activity of the adrenergic system in salivary glands (Davis & Granger, 2009; Green & Bavelier, 2003).

In addition, research findings suggest that the type of stress is associated with concentration, which can have positive or negative effects (Boot, Kramer, Simons, Fabiani, & Gratton, 2008; LaBar & Cabeza, 2006; Weber, Ritterfeld, & Mathiak, 2006). In video games, stress varies by the game style. For instance, the logic stress is a type of stress under which the person only searches for logical solutions to handle the stressor and the situation. Under this type of stress, the person feels no temporal and spatial constraint and can travel the path to the goal at any time. In addition, no destructive pressure is put on the person, and the person can use his or her knowledge and capabilities to attain the goals peacefully and even reinforce his or her capabilities (LaBar & Cabeza, 2006; Szubert & Jaśkowski, 2014). The main stress factor involved in the development of the limit stress is restraint, and its evident examples are temporal restraints. Under this type of stress, the person needs to accomplish the tasks quickly and carefully to handle the situation. To solve the problem, the person not only must pay adequate attention but also does need to have the ability to make temporal and spatial predictions and function quickly to coordinate the neural and muscular (sensory-motor) systems (Boot et al., 2008; Gracia-Bafalluy & Noel, 2008). Fear stress is undoubtedly the type of stress we have all experienced. Under this type of stress, the activity of the neuroendocrine system is at its peak, and thus the characteristics of the vague type of stress are evident in this type associated with recognizable signs and symptoms. It deprives the affected person of the ability to decide accurately and duly. Moreover, the affected person loses its concentration on the environment and fails to function satisfactorily (Burdo, 2014; Weber et al., 2006). Interactive stress is a type of stress associated with interaction with the surrounding elements in video games. Under this type of stress, the affected person experiences stress due to his or her presence in the environment, the sensory inputs (including the visual, auditory, olfactory, and tactile inputs), and his or her previous experiences and memories (Ballard & Wiest, 1996; LaBar & Cabeza, 2006).

EEG signals reflect the electric activities of the brain. These signals carry useful information on the performance of the brain, and thus are widely used in the fields of medicine, pathological conditions, mind engineering, and cognitive markers. This method is widely used by different researchers in various fields to noninvasively assess the variations of brain signals (Ballard & Wiest, 1996; Burdo, 2014). As a powerful means of demonstrating brain activity, EEG signals play a major role in most studies on brain. Hence, the ability to measure and record EEG signals that are free of pollution is an important goal of these studies. Perhaps an EEG signal is the only type of data that provides online and direct information on the condition of the brain and its activity (Burdo, 2014; Weber et al., 2006). Since stress is not always destructive and its effects depend on its type, this study was conducted to examine the different types of stress with regard to the style of video games.

## 2. Methods

A total of 80 male volunteers aged between 18 and 30 years enrolled in this research project. The study questionnaire gathered the relevant data about personal characteristics of the players, the games of interest, game type, how much do they play per day, and so on. The volunteers were grouped into four (each 20 members). In this research, four video games namely the Runner game, Fear game, Excitement game, and Puzzle game, were investigated. Each group played one type of the games. The saliva samples of all participants were collected before and after playing the games using 10-mm falcon tubes, which were stored at −20°C in a freezer. On the day of experimentation, the samples were liquefied at room temperature and centrifuged at 3000 rpm for 5 minutes. Afterwards, 20 μL of each sample was isolated for the test, and the human cortisol ELISA kit (Diagnostics Biochem Canada Inc.,) was used to measure the level of salivary cortisol. Also, Pars Azmun kit was used to measure the levels of salivary α-amylase. The brain waves of the players were recorded and analyzed online using an Emotiv device. A total of 14 electrodes were placed on different locations of participants’ heads from the beginning to the end of the games to record the changes of brain waves. To identify the differences, first, the EEG signals of the participants’ brains were recorded. Afterwards, the data were filtered and the noises were omitted. The necessary features were extracted and classified using the machine learning algorithms. In the end, the data were analyzed and the algorithms were tested. The obtained data were analyzed in Matlab and R.

### 2.1. Study video games

#### 2.1.1. Puzzle game

A Puzzle game generally improves concentration, memory, and the harmony between the hands and the brain. This game is based on the mathematical sum function. Interestingly, this type of game has gained extensive popularity after a short period of time, because its immense allure entertains the player for hours. Stress is caused by the fear of inability to function.

#### 2.1.2. Runner game

The symphony of tiles makes the user calm and enables him or her to avoid addiction to the game. This game is classified as a musical game. Another source of attraction of this game is the excitement caused by the rapid and simultaneous movement of the fingers and the eyes, which results in the composition of a pleasant song. The cause of stress in this game is time limitations.

#### 2.1.3. Fear game

Many video games released in the fear and horror category claim to induce fear in the player. Some of these games show disgusting scenes, some others try to frighten the player with the aid of psychological stresses, and a group of games uses a combination of these factors. The key to winning this game is to frighten the player with the aid of sound effects. You will be frightened when you play this game but the fear is mainly caused by the mysterious sounds rather than the disgusting faces of the characters. This type of fear is known as the fear of the unknown and is considered a psychological type of fear.

#### 2.1.4. Excitement game

It is a violent and highly exciting game in which the player fights all of his or her rivals and beats them to win the game. You must get used to the bloodbath and the torn apart body parts you will see during the game. However, the blood, flesh pieces, and crushed bones are the elements of a very detailed and entertaining fighting game that amaze you from the moment you start to play. You will fight characters that fight quickly and thoughtlessly in a highly dynamic detailed environment.

### 2.2. Data analysis

The research data were expressed as Mean±SD (Mean±SEM) values. The non-parametric Wilcoxon test method was used to determine the differences between the groups. The significance level was also set at <0.05.

## 3. Results

**Figure 1:**
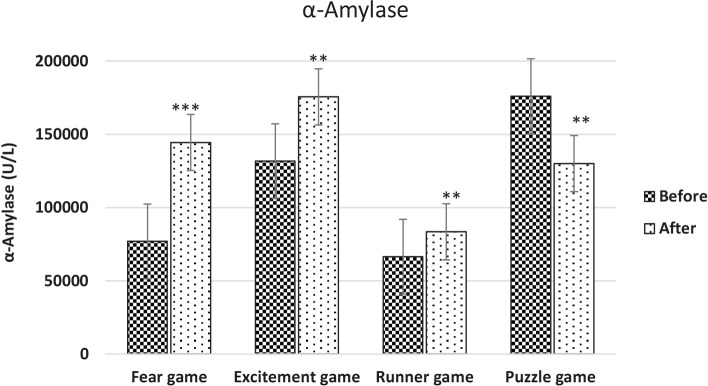
The variations of the mean concentrations of the salivary α-amylase in the players that increased significantly after playing the four video games

The research findings indicate that the variations of the salivary α-amylase concentration decreased significantly after playing the Puzzle game type (P<0.001) (Figure 1). In addition, it was found out that the concentrations of the salivary α-amylase increased after playing the Runner game type (P<0.001) (Figure 1). It was also found that the variations of the salivary α-amylase escalated significantly after playing the Fear game type (P<0.0001) (Figure 1).

**Figure 2:**
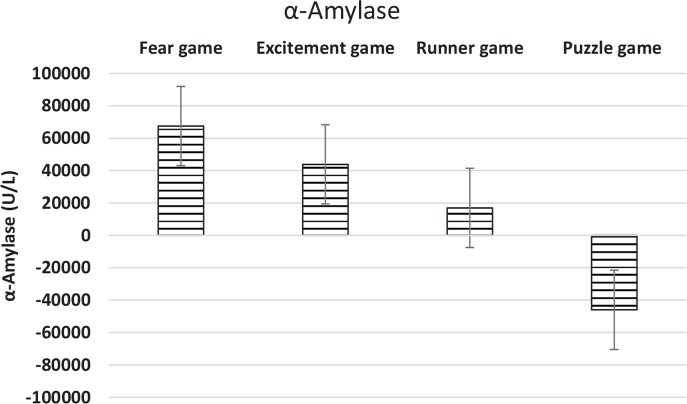
The mean variations of the concentration of α-amylase after playing the four games The largest change was caused by the Fear game.

Finally, the changes of the variations of the salivary alpha-amylase increased significantly after playing the Excitement game type (P<0.001) (Figure 1). The mean variations of the concentration of α-amylase are depicted in Figure 1 and the percentages of the mean variations of the salivary α-amylase are presented in Table 1. According to Figure 2 the largest change was caused by the fear game.

**Table 1:** The percentage of the variations of the mean concentration of α-amylase after playing the games

	**Difference**	**Percentage 1**	**Percentage 2**
Fear game	67510.71	87.79429	46.75024
Excitement game	43835.88	33.29807	24.98016
Runner game	16949.6	25.4969	20.31676
Puzzle game	−45998.3	−26.1317	−35.376

**Figure 3:**
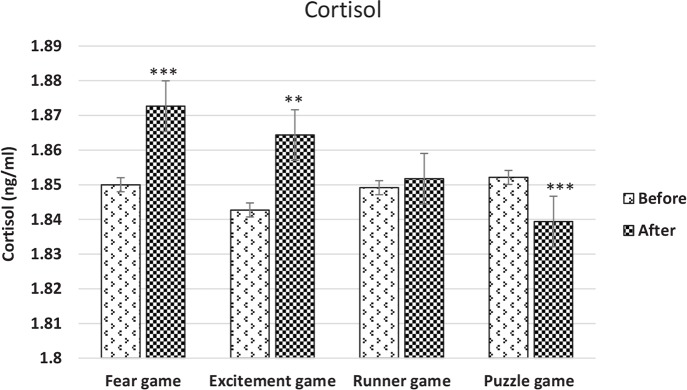
The variations of the mean concentration of the salivary cortisol in the participants increased significantly after playing the four games

According to the research findings, the variations of the concentration of the salivary cortisol decreased significantly after playing the Puzzle game type (P<0.001) (Figure 3). The variations of the concentration of the salivary cortisol rose significantly after playing the Runner game type (Figure 3). The variations of the concentration of the salivary cortisol escalated significantly after playing the Fear game type (P<0.0001) (Figure 3).

**Figure 4:**
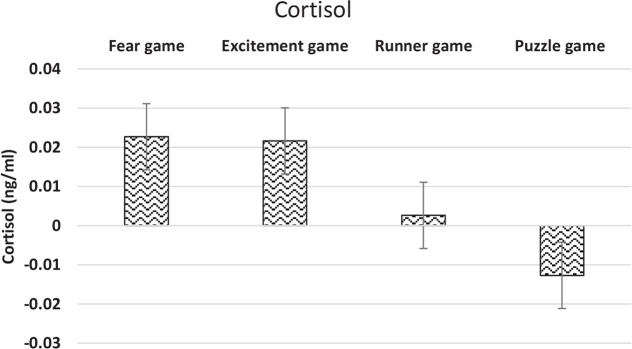
The mean variations of the concentration of cortisol after playing the game showed that the highest change was caused by the fear game

Finally, the variations of the concentration of the salivary cortisol escalated significantly after playing the Excitement game type (P<0.001) (Figure 3). In addition, the mean variations of the salivary cortisol after playing the four games and the percentages of the mean variations of the salivary cortisol are shown in Figure 4 and Table 2, respectively. Our investigations showed that the activity of the right forehead hemisphere of people experiencing social anxiety or people exposed to social threats is higher.

**Table 2:** The percentages of the variations of the mean concentration of cortisol after playing the games

	**Difference**	**Percentage 1**	**Percentage 2**
Fear game	0.022676	1.225727	1.210885
Excitement game	0.021607	1.172535	1.158946
Runner game	0.002611	0.141184	0.140985
Puzzle game	−0.0127	−0.68574	−0.69047

**Figure 5:**
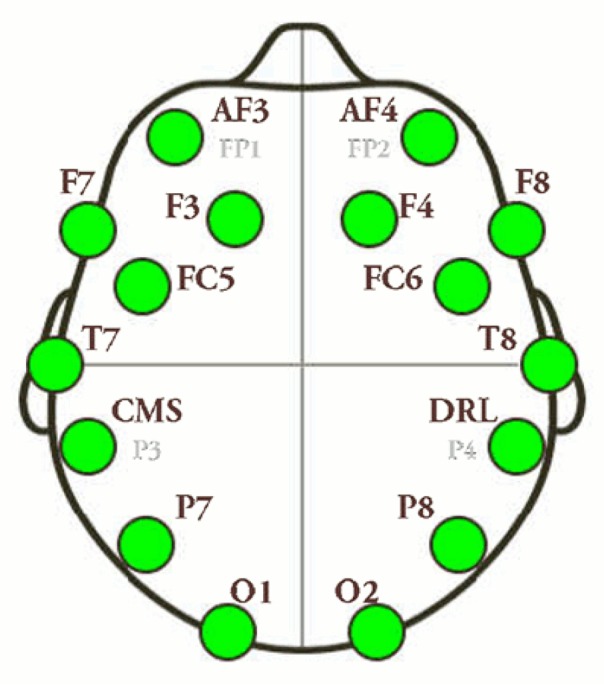
The positions of the electrodes on the participants’ heads

Therefore, based on these findings and neurofeedback sources, the method selected for analyzing and determining the level of stress must show the ratio of the activity of the two brain hemispheres in the forehead. Figure 5 depicts the positions of the electrodes on the heads of the players. Note that the relatively higher activity of the right hemisphere as compared to the left hemisphere reflects the relatively higher α activity in the left hemisphere. Moreover, the signals of different people were analyzed with regard to the mentioned stress type.

**Figure 6:**
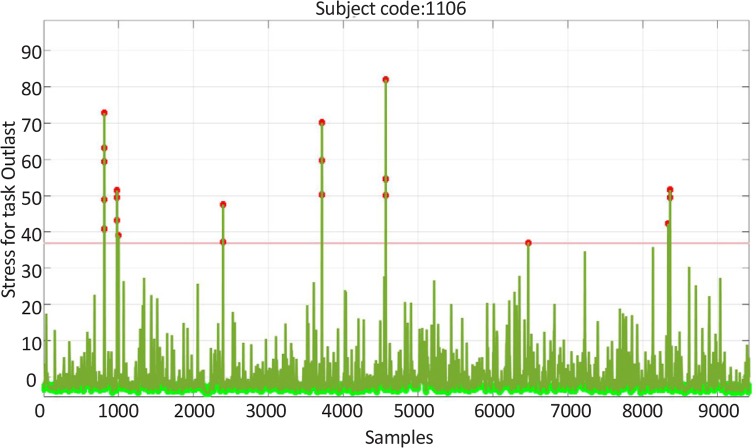
The changes of the stress feature of the brain signals of a participant after playing the fear game

**Figure 7:**
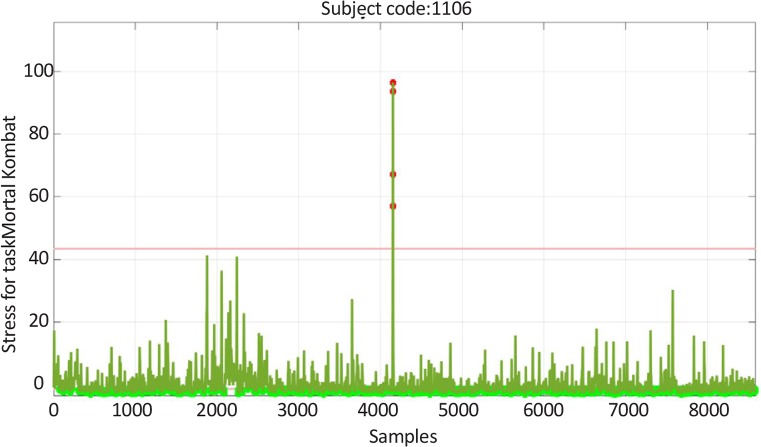
The changes of the stress feature of the brain signals of a participant after playing the Excitement game

**Figure 8:**
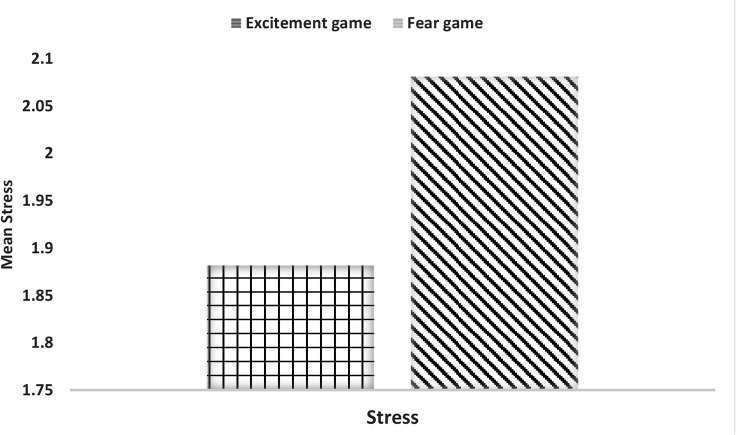
The levels of the stress indicator in two different groups

Figures 6 and 7 are indicative of the level of fear (the stress performance of the brain signals) in two individuals who played the Fear game and Excitement game under similar test conditions. It is worth stating that these diagrams are zero mean diagrams and the horizontal direction is a temporal direction with 0.0625 second increments (Lupien & Lepage, 2001; Rosenbloom et al., 2012). Figures 6 and 7 shows the mean stress of the participants or the stress performance of the brain signals, which equals the ratio of the alpha power of the frontal left hemisphere to the frontal right hemisphere, in different time points. The mean results of each player of each group are also depicted in the bar diagram No. 1 (Figure 8).

## 4. Discussion

The players of video games are different with respect to their age, gender, nationality, and time zone. The importance of video games lies in their effect on the central nervous system of the gamers and their ever-increasing number. The different styles of video games attract different users and have different effects. Video games use moving pictures and exciting sounds to bring a world of excitement to the fans. This attraction brings the user’s soul and body to a world of imagination and makes him or her feel like a hero of the story. These effects use various techniques and tricks to affect the thoughts and minds of the players to the extent that they think they are a part of a whole (Boot et al., 2008; David, Harris, & Bley Jr, 1983; Davis & Granger, 2009; LaBar & Cabeza, 2006; Weber et al., 2006). Related research suggests that action video games can affect the visual short-term memory, spatial understanding, multi-tasking, and some of the aspects of performance (Boot et al., 2008; LaBar & Cabeza, 2006).

Research results also indicate that cortisol is the most important hormone secreted by the adrenal gland cortex of humans in response to stress. When the human brain considers a factor a stress (or risk) factor, amygdala is activated and triggers the parontotrichular nucleus of the hypothalamus. As a result, corticotropin-releasing hormone is released from the neurons of this nucleus into the blood. This hormone affects the secreting cells and causes the release of adrenocorticotropin from these cells into the blood (Cardinal, Parkinson, Hall, & Everitt, 2002; Fuster, 1988; Gray, Braver, & Raichle, 2002; Lupien & Lepage, 2001; Smeets et al., 2008). In addition, the adrenal sympathetic stress axis, as the second axis, responding to stress in the human body, increases the secretion of ptyalin into the saliva by stimulating the sympathetic system and increasing the norepinephrine hormone. The variations of the salivary ptyalin are the measures of cortisol and α-amylase. According to the investigation, chronic stress leads to mental dysfunction, whereas the acute stress reinforces mental abilities such as attention, processing speed, and the decision-making power (Davis & Granger, 2009; Maheu et al., 2005). Moreover, the effects of stress on the cognitive performance of humans depend on the effective dose of cortisol (LaBar & Cabeza, 2006; Pham, & Tran, 2012; Ramirez & Vamvakousis, 2012).

The research results also reveal that the levels of cortisol and concentration of α-amylase decrease significantly in the participants who played the Puzzle game. This game is designed based on logic and problem-solving skills, and due to the activation of the decision-making parts of the brain (including the prefrontal lobe) the stress system is deactivated and the levels of cortisol and α-amylase decrease. This type of stress can be defined as the logic stress (Szubert & Jaśkowski, 2014). The analyses of the Runner game showed that the concentration of cortisol increased slightly after playing this game. The increase in stress equals the circumvention of the time limitation, which increases the activity of the stress system and induces fear to overcome this limitation. This time limitation is thought to have positive effects, increases human’s speed and reinforces the eye-hand coordination. This kind of stress is known as the limit stress (Gracia-Bafalluy, & Noel, 2008).

The other findings of this research show that the levels of cortisol and α-amylase increase significantly in the players of Fear type game. Since this game is a horror game it can be destructive in the long run because the players are constantly feeling fear and stress, the adrenal gland and the hypothalamus-pituitary axis are always ready to function, and cortisol is released abnormally. These endless activities impair human’s cognitive functions such as concentration, attention, decision-making, and memory in the long run. This type of stress is known as the fear stress (Burdo, 2014). The increase in the unnatural secretion of cortisol impairs the glutamate system, destroys the nervous synapses, and results in the impairment of memory, learning, and emergence of anger, violence, or depression. The increase in the concentration of extracellular glutamate and the excessive activity of the ionotropic receptors cause toxicity, which damages the nerve cells and the hippocampus synapses (Krawczyk, 2002; Smeets et al., 2008; Yu, 2016).

The results of studies on the violent Excitement game showed that the levels of cortisol and α-amylase of the players increased significantly after playing this game. This game is formed of a set of elements that are not superior to one another, and thus the resulting stress cannot be attributed to a certain feature of the game. This is because stress is caused by violent and frightening visual and sonic elements as well as timely decisions and logic of the player. In other words, this game induces interactive stress (Ballard & Wiest, 1996). Since the NMethyl-D-Aspartate (NMDA) receptors play a major role in transmitting the stimulating messages, plasticity, and neural destruction of the central nervous system, the excessive activity of these receptors leads to the generation of large amounts of neurotoxic substances that demolish the nerve cells and synapses. The physiological effects of the damage are demonstrated in the form of unwanted angry and violent behavior and depression and a decrease in the cognitive readiness of the individual (Ballard & Wiest, 1996; Fox, 2011).

Recently, stress is defined a mental state that affects human brain and body. According to this definition, by playing the Puzzle and Runner game, the players’ stress-fear system is almost deactivated due to the activity of the decision-making, attention, and concentration zones of the brain (which include the prefrontal area and frontal lobes). In addition, as seen in the provided diagrams, the deactivation of the fear-stress system decreased significantly after playing Puzzle game and slightly after playing the Runner game. This difference is caused by the described stress. In other words, the style-wise results of this research proved that the elements of these games had no destructive effect on the stress-fear systems of the participants. The examinations of the Excitement game and Fear game showed that the level of α-amylase (as the marker of fear) and cortisol (as the market of stress) changed significantly.

The escalation (%) of α-amylase concentration was considerably higher in the players of the Fear game than the players of the Excitement game. This finding reflects the differences between the game contents. After playing these games the concentrations of cortisol increased, but the percentage of variations was higher in the Fear game. However, these differences were not considerable as compared to the variations of the α-amylase levels. The results obtained by recording the brain waves of the players mainly indicated the development of fear after playing the Fear game. In addition, the analysis of the brain signals of the players who played the Excitement game showed high levels of stress. Figure 8 shows that the Fear game players had a larger stress performance index compared to Excitement game players. In general, the type of stress depends on the game style, and the resulting stress may reinforce the cognitive elements or destroy them, but more research is required to clarify the effects.

## Ethical Considerations
